# *Ascodipteron* (Diptera, Nycteribiidae, streblid grade) from China: a new species from the lesser brown horseshoe bat, *Rhinolophus stheno*

**DOI:** 10.3897/zookeys.1273.183551

**Published:** 2026-03-23

**Authors:** Haoran Sun, Liang Ding, Dong Zhang, Thomas Pape

**Affiliations:** 1 School of Ecology and Nature Conservation, Beijing Forestry University, Qinghua East Road 35, Beijing 100083, China Cheeloo College of Medicine, Shandong University Jinan China; 2 Department of Pathogenic Biology, School of Basic Medical Sciences, Cheeloo College of Medicine, Shandong University, Jinan, China Science Faculty, University of Copenhagen Copenhagen Denmark; 3 Natural History Museum of Denmark, Science Faculty, University of Copenhagen, 2100 Copenhagen, Denmark School of Ecology and Nature Conservation, Beijing Forestry University Beijing China

**Keywords:** *
Ascodipteron
*, bat fly, endoparasite, identification key, mitochondrial gene segments, morphology, taxonomy

## Abstract

*Ascodipteron euryale***sp. nov**. is described from Yunnan Province, China, based on dealate neosomic females extracted from the base of the ear of the lesser brown horseshoe bat, *Rhinolophus stheno* K. Andersen, 1905; it is the first record of a species of *Ascodipteron* Adensamer, 1896 from this bat. Morphological evidence is further supported by molecular data from the cytochrome *b* (*Cytb*) and cytochrome *c* oxidase subunit I (*COI*) genes. A detailed comparison of the new species with related species is provided, and the new species is accommodated in the most recent key to the world species of *Ascodipteron*.

## Introduction

Bat flies of the genus *Ascodipteron* are restricted to the Eastern Hemisphere, and the genus contains about 17 valid species found in tropical and subtropical areas (Hastriter 2007; Sun et al. 2021, 2022). Females are exclusively endoparasitic, and after a short host-seeking period, they shed wings and legs to live embedded in the skin of bats as highly modified neosomes. Embedded females are not uncommon on certain species of the bat genera *Emballonura*, *Hipposideros* and especially *Miniopterus* and *Rhinolophus*, and many *Ascodipteron* spp. show a narrow host range and specific preference for location on the host (Hastriter 2007).

In China, five species of *Ascodipteron* have been documented (Hastriter 2007; Sun et al. 2021, 2022; Wang et al. 2022). In the present study, we describe a new species of *Ascodipteron* from Yunnan with comprehensive morphological documentation and sequence data for the cytochrome *b* (*Cytb*) and cytochrome *c* oxidase subunit I (*COI*) genes. The new species is incorporated into a key to females of the recognized species of the genus.

## Material and methods

### Specimen collection and preparation

Five bat flies were collected from a colony of the lesser brown horseshoe bat, *Rhinolophus stheno* K. Andersen, 1905 in August 2022, roosting in a large cave in China, Yunnan Province, Pu’er City, Simao District, Liushun Town, Xianren Cave, 2428 m a.s.l., (22°36'13.96"N, 100°42'47.01"E). Entire female ascodipterine bat flies (neosomes) were removed with forceps without hurting the host.

All specimens were preserved in 95% ethanol and deposited at the Museum of Beijing Forestry University, Beijing, China (MBFU).

### Specimen imaging, measurements, and terminology

Z-stack photographs were acquired with a Visionary Digital Imaging System using a Canon EOS 7D (Canon, Inc., Tokyo, Japan) and were stacked with Zerene Stacker software. Photographs were taken with a Canon EF 100 mm f/2.8L IS USM and MP-E 65 mm f/2.8 1–5× lenses attached to a Canon 5D Mark IV SLR camera. Images and plates were processed on a standard Windows 10 platform using Adobe Photoshop 2021 (Adobe Systems, Inc., San Jose, CA, USA). Measurements and terminology follow Sun et al. (2022) and Cumming and Wood (2017).

### DNA sequence analysis

The whole body of one specimen (BFU-2476) was used to extract total genomic DNA using the TIANamp Genomic DNA Kit (Tiangen, Beijing, China). After extraction, the remaining body parts (head, thorax and transparent abdomen) were cleaned with demineralized water and retained as vouchers, deposited in the Museum of Beijing Forestry University, Beijing, China (MBFU). The mitochondrial cytochrome *c* oxidase subunit I (*COI*) gene and the mitochondrial cytochrome *b* (*Cytb*) gene were amplified, sequenced and edited following Sun et al. (2022), except that the *Cytb* gene was amplified using the primer pair B2 (forward: 5'-TGA TGA AAY YTT GGA TCA TTA-3') and B1.1 (reverse: 5'-AAA TAT CAT TCT GGT TGA ATA TG-3') as in Alcantara et al. (2022). All sequence data were uploaded to GenBank.

The same 11 sequences of the cytochrome *b* gene (*Cytb*) and 13 sequences of the cytochrome *c* oxidase subunit I (*COI*) used by Sun et al. (2022) were downloaded from GenBank and combined with the newly generated sequences (Table 1). All sequences were aligned using Muscle as implemented in Mega X (Kimura 1980; Kumar et al. 2018), and nucleotide sequence divergences were calculated using the Kimura 2-parameter (K2P) model in Mega X.

**Table 1.** Specimens with GenBank accession numbers. Superscript numbers following the binominal names indicate different specimens of the same species. Sequences downloaded from GenBank marked by asterisks. ”—” indicates no data.

Species	GenBank Accession Numbers	
	*Cytb*	*COI*
*Ascodipteron euryale* sp. nov.	PX645105	PZ167828
*Ascodipteron guoliangi* ^1^	—	OP900074
*Ascodipteron guoliangi* ^2^	OP903228	OP900075
*Ascodipteron phyllorhinae* ^1^	—	OP900076
*Ascodipteron phyllorhinae* ^2^	—	OP900077
*Ascodipteron phyllorhinae* ^3^	OP903229	OP900078
*Ascodipteron phyllorhinae* ^4^	OP903230	OP900079
*Ascodipteron phyllorhinae* ^5^	DQ133149	—
*Ascodipteron sanmingense* ^1^	OP903231	OP900080
*Ascodipteron sanmingense* ^2^	—	OP900081
*Ascodipteron sanmingense* ^3^	OP903232	OP900082
*Ascodipteron sanmingense* ^4^	OP903233	—
*Ascodipteron sanmingense* ^5^	—	OP900083
*Ascodipteron sanmingense* ^6^	—	OP900084
*Ascodipteron sanmingense* ^7^	MW822598	—
*Ascodipteron speiserianum* ^1^	OP903234	OP900085
*Ascodipteron speiserianum* ^2^	OP903235	OP900086
*Ascodipteron* sp. ^1^	DQ133154	—

## Results

### Class Insecta Linnaeus, 1758

**Order Diptera Linnaeus, 1758**

**Superfamily Hippoboscoidea Samouelle, 1819**

**Family Hippoboscidae Samouelle, 1819**

#### Genus Ascodipteron

Taxon classificationAnimaliaDipteraHippoboscidae

Adensamer, 1896

10CA866D-963E-5515-BEC8-86879B9866DB

##### Type species.

*Ascodipteron phyllorhinae* Adensamer, 1896, by monotypy.

#### Ascodipteron euryale sp. nov.

Taxon classificationAnimaliaDipteraHippoboscidae

11D938D5-4B8E-535A-BD4F-5F91BAB08DBA

https://zoobank.org/6879E2CA-4470-49B6-A67E-9DF77AA6830B

Figs 1, 2, 3, 4

##### Diagnosis.

Mesosternum with rounded postero-lateral margins (Fig. 3C, 4A), which is not found in any other species of *Ascodipteron*.

**Figure 1.**
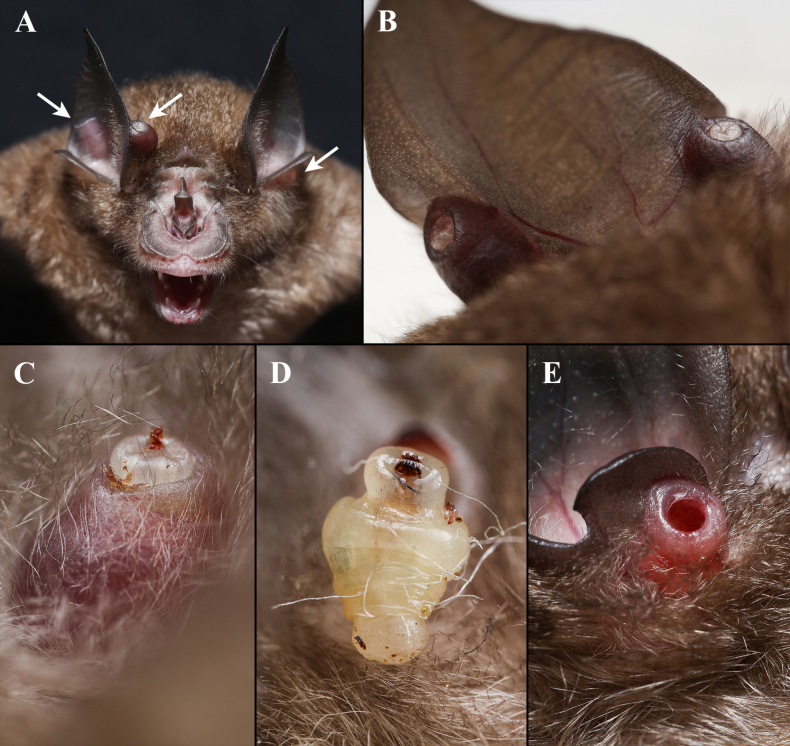
*Ascodipteron euryale* sp. nov. and its host *Rhinolophus stheno*. **A, B**. Roosting host with neosomes in ears (arrows); **C**. Neosome terminalia protruding from host tissue; **D**. Neosome plucked from host tissue; **E**. Hard shell (fibrous host cyst) remains on the host after the removal of neosome.

**Figure 2.**
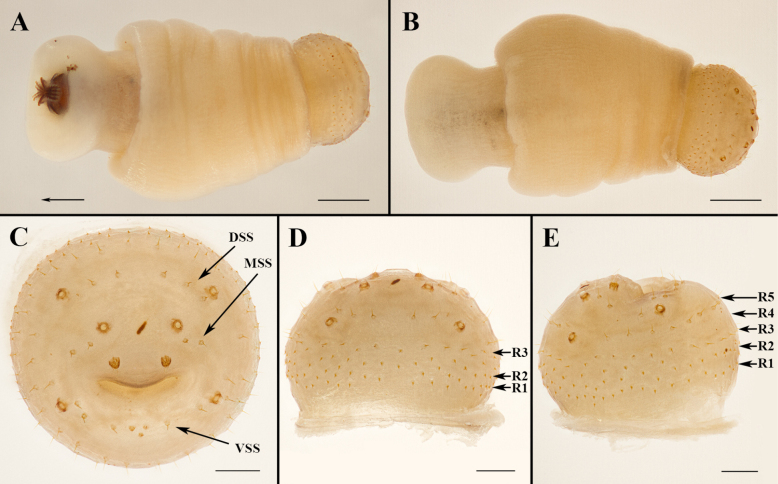
*Ascodipteron euryale* sp. nov., ex. *R. stheno*. **A, B**. Whole neosome (head and thorax fully withdrawn, arrow indicates direction of the head) (BFU-2474), ventral view (**A**), and dorsal view (**B**); **C–E**. Terminalia, posterior view (**C**), dorsal view (**D**) and lateral view (**E**). Abbreviations: DSS = dorsal spiracular setae; MSS = medial spiracular setae; VSS = ventral spiracular setae; R1–5 = abdominal setae arranged roughly into annular rows comprised of variable types of setae, R1 the proximal and R5 the distal row. Scale bars: 0.5 mm (**A, B**); 0.2 mm (**C, D**).

**Figure 3.**
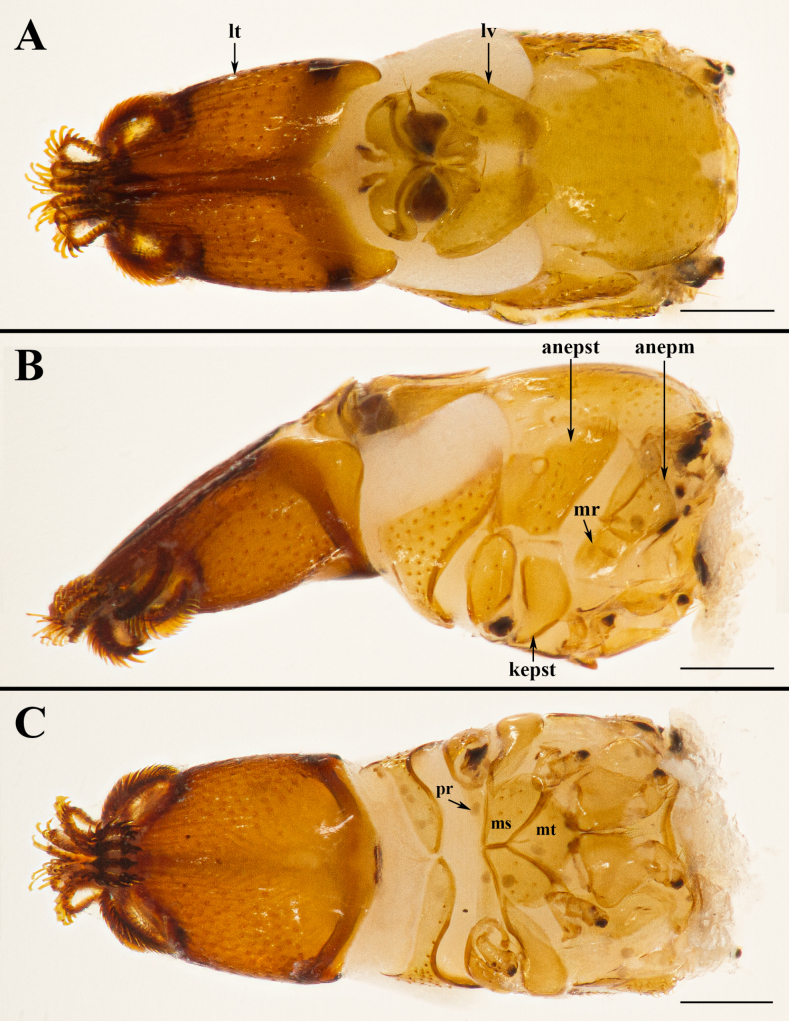
*Ascodipteron euryale* sp. nov., ex. *R. stheno*, head and thorax (BFU-2474). **A**. Dorsal view; **B**. Lateral view; **C**. Ventral view. Abbreviations: anepm = anepimeron; anepst = anepisternum; kepst = katepisternum; lt = labial theca; lv = lateral vertex; mr = meron; ms = mesosternum; mt = metasternum; pr = prosternum. Scale bar: 0.2 mm.

**Figure 4.**
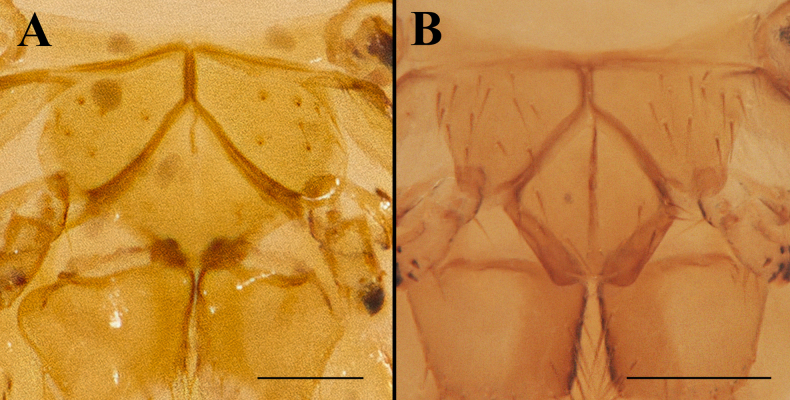
Mesosternum and metasternum, ventral view. **A**. *Ascodipteron euryale* sp. nov. (BFU-2474); **B**. *A. guoliangi* Sun, Ding, Pape & Zhang, 2022 (BFU-2437). Scale bar: 0.2 mm.

##### Etymology.

The name refers to Euryale, one of three sisters, the Gorgons, in Greek mythology, specifically the three female monsters, Stheno, Euryale, and Medusa, who could turn anyone looking at them into stone (Grimal 1996). Euryale and Stheno were immortal, which is a fitting expression of our hope that the deep-rooted association between *Rhinolophus stheno* and *Ascodipteron euryale* sp. nov. will continue long into the future.

##### Type material.

***Holotype***. China – **Yunnan Province** • ♀ (dealate); Pu’er City, Simao District, Liushun Town, Xianren Cave; 22°36'13.96"N, 100°42'47.01"E; 2428 m a.s.l.; 30 Aug. 2022; H.R. Sun and L. Ding leg.; ex. *Rhinolophus stheno*, in the ear; BFU-2473; MBFU. ***Paratypes***. China – **Yunnan Province** • 4 ♀ (dealate); same data as holotype; BFU-2474, BFU-2475-1, BFU-2475-2, BFU-2476; MBFU.

##### Description.

**Female**. Head. Labial theca slightly longer than wide; posterior margin concave dorsally, convex ventrally. Labial theca dorsally with c. 130+ pigmented, peg-like, spiniform setae and ventrally with c. 140+ similar setae. Gena with c. 40 irregularly scattered peg-like setae, anterior part bare in ventral view, posterior margin concave laterally, convex medially. Arista with multiple fine branches, basal antennal segment with single long seta. Lateral vertex twice as long as wide, with concave anteromedial margin; adorned with 23–28 long, thin setae on each side.

Thorax. Scutum with numerous long setae, but devoid of setae along mid-line. Anepisternum (mesopleuron) with 0–3 setae anterior to the large, round spiracle; setae posterior to spiracle of two varieties: 2–6 short, peg-like, spiniform setae (lower one-third); 17–26 longer, slender setae (upper two-thirds). Anepimeron (pteropleuron) with 8–11 peg-like setae and 3–11 long, slender setae unevenly distributed. Meron (hypopleuron) and katepisternum (sternopleuron) without setae. Prosternum without setae, mesosternum rounded on postero-lateral margin, with 6–8 peg-like or slender setae, metasternum with 1–2 slender setae.

****. Abdominal setae arranged roughly into annular rows, conventionally referred to as R1–5, with R1 the proximal and R5 the distal row. R1 and R2 with short, thick setae forming irregular rings that sometimes look like 3 rows; R3 and R5 with longer and more slender setae, R4 absent. R1, R2 and R3 incomplete ventrally and R5 only found ventrally; 8 long ventral spiracular setae (VSS) in arching row or grouping between spiracles 7; 2 medial spiracular setae (MSS) with paired symmetrical groups situated between spiracles 6 and 7 on each side; 6 long dorsal spiracular setae (DSS) in single arching row between spiracles 5. Cercus with a few minute setae.

***Dimensions***. Head and thorax: total length 1.5 mm; labial theca, length: 0.6 mm, width: 0.5 mm; terminalia, diameter: 1.1 mm; neosome, length: 3.4 mm.

**Male**. Unknown.

##### Distribution.

Oriental—China (Yunnan).

##### Remarks.

*Ascodipteron euryale* sp. nov. is most similar to *A. guoliangi* Sun, Ding, Pape & Zhang, 2022, and it will run to couplet 16 in the identification key to dealate ascodipterine females proposed by Sun et al. (2022). It can be incorporated in that key as follows.

### Modified key to dealate ascodipterine females (couplets 1–15 as in Sun et al. (2022); bat hosts and distribution are given in square brackets)

**Table:** 

16	First abdominal annular row (R1) present; fourth abdominal annular row (R4) absent	**18**
–	First abdominal annular row (R1) absent; fourth abdominal annular row (R4) present [*Rhinolophus* spp., Africa.]	***A. brevior* Maa, 1965**
17	MSS comprised of one seta. R1 and R2 absent [*M. schreibersi*, Africa]	***A. theodori* Maa, 1965**
–	MSS lacking setae. R1–R3 present, with short, triangular spines [*M. schreibersi*, Africa]	***A. minor* Theodor, 1973**
18	Labial theca dorsally with c. 50+, peg-like, spiniform setae and ventrally with 100+ uniform setae; mesosternum with postero-lateral part more angulate, with entire mesosternal sclerite almost trapezoidal (Fig. 4B) [*Coelops frithii*, in elbow pit where upper arm meets forearm, SE China]	***A. guoliangi* Sun, Ding, Pape & Zhang, 2022**
–	Labial theca dorsally with c. 130+ pigmented, peg-like, spiniform setae and ventrally with c. 140+ similar setae; mesosternum with postero-lateral part forming a large, gently rounded lobe (Fig. 4A) [*Rhinolophus stheno*, at base of ear, in hard shell (fibrous cyst), SE China]	***A. euryale* sp. nov**.

### Molecular results

A 675 bp *Cytb* fragment and a 554 bp *COI* fragment were obtained for *A. euryale* sp. nov. (Table 1). Pairwise comparisons with other species of *Ascodipteron*, for which relevant data are available, yielded average genetic divergences in the range 13.00%–14.93% for *Cytb* (Table 2) and 7.65%–8.88% for *COI* (Table 3).

**Table 2.** Pairwise differences of mitochondrial cytochrome *b* gene (*Cytb*) sequences between species based on Kimura 2-parameter.

		1	2	3	4	
1	*Ascodipteron euryale* sp. nov.					
2	*Ascodipteron guoliangi*	0.1354				
3	*Ascodipteron phyllorhinae*	0.1364	0.0526			
4	*Ascodipteron sanmingense*	0.1300	0.0951	0.0782		
5	*Ascodipteron speiserianum*	0.1493	0.1276	0.1114	0.1184	
6	*Ascodipteron* sp.	0.1431	0.1203	0.1068	0.1173	0.0895

**Table 3.** Pairwise differences of cytochrome *c* oxidase subunit I gene (*COI*) sequences between species, based on Kimura 2-parameter.

		1	2	3	4
1	*Ascodipteron euryale* sp. nov.				
2	*Ascodipteron guoliangi*	0.0765			
3	*Ascodipteron phyllorhinae*	0.0785	0.0427		
4	*Ascodipteron sanmingense*	0.0886	0.0657	0.0552	
5	*Ascodipteron speiserianum*	0.0888	0.0825	0.0766	0.0795

## Discussion

The unique shape of the mesosternum will separate *A. euryale* sp. nov. from all other species of this genus, and its status as a separate species is strongly supported by the molecular data with ample genetic divergence from those congeners for which comparable molecular data exist (Table 3).

Species of *Rhinolophus* often appear as hosts for females of *Ascodipteron*. However, the present record of *Ascodipteron euryale* sp. nov. is the first documentation of *Ascodipteron* from the lesser brown horseshoe bat, *Rhinolophus stheno* K. Andersen.

Five species of *Ascodipteron* are known as neosomes from the ear of their host: *A. brevior* (base of ear), *A. lophotes* Monticelli, 1898 (base of ear), *A. rhinolophi* Jobling, 1958 (base of ear), *Ascodipteron sanmingense* Sun, Ding, Yan, Pape & Zhang, 2021 (base of ear and lower jaw area) and *A. speiserianum* Muir, 1912 (base of ear/ear pinna) (Maa 1965; Hastriter 2007). The neosomes are all recorded from the “base of ear”, and like *A. euryale* they are probably located at the junction of the root of the ear and the head proper, with the proboscis directed into the head tissue and the remaining part of the neosome at most partly embedded in the cartilaginous part of the ear. Hastriter (2007) gave “presumably … under skin at base of ear” for the lectotype of *A. speiserianum* and “ear pinna” for the neotype of *A. australiansi* Muir, 1912 (= *A. speiserianum*), but for the latter it was also given as “base of ear”, which is in agreement with Maa (1971), who mentioned ten *A. speiserianum* neosomes from the base of the ears of *Miniopterus* spp in Australia.

*Ascodipteron euryale* sp. nov. neosomes were found embedded at the base of the host ear pinna or tragus and within a hard or fibrous cyst or shell, making it difficult to extract the specimens. Hastriter and Bush (2006) and Hastriter (2007) described a hard shell for *Maabella stomalata* Hastriter & Bush, 2006, which embeds in the leading wing margin and over the bones of the phalanges, often so closely associated with the underlying bony tissue that the hard shell appears to be calcified extensions of the bone. The hard shells of *A. euryale* sp. nov. are not formed by proliferation of ear cartilage, as they are fully separated from this. Hastriter (2007, fig. 1E) provided a photograph of a cyst left from an extracted neosome of *A. phyllorhinae*, mentioning that a fibrous cyst wall surrounding the neosome usually formed as a host reaction to the embedded parasite. According to our collection in the field of *A. guoliangi*, *A. phyllorhinae*, *A. sanmingense*, and *A. speiserianum* (H. Sun pers. obs.), no hard shell was formed, and neosomes were covered in soft skin and, therefore, easy to pull out. The cause of the formation of hard or fibrous cysts or shells requires further study.

## Supplementary Material

XML Treatment for Ascodipteron

XML Treatment for Ascodipteron euryale

## Additional information

### Use of AI

No use of AI was reported.

### Author ORCIDs

Haoran Sun https://orcid.org/0000-0003-1785-4160

Liang Ding https://orcid.org/0009-0005-3938-4544

Dong Zhang https://orcid.org/0000-0001-6427-7867

Thomas Pape https://orcid.org/0000-0001-6609-0609

### Data availability

All of the data that support the findings of this study are available in the main text.

## References

[B1] Alcantara DMC, Graciolli G, Junior MA, Toma R, Nihei SS (2022) Biogeographical events, not cospeciation, might be the main drivers in the historical association between *Noctiliostrebla* species (Streblidae) and their bulldog bat hosts. Biological Journal of the Linnean Society 137(4): 583–602. 10.1093/biolinnean/blac097

[B2] Cumming JM, Wood DM (2017) Adult morphology and terminology. In: Kirk-Spriggs AH, Sinclair BJ (Eds) Manual of Afrotropical Diptera. Vol. 1. Suricata 4, SANBI Publications, Pretoria, 89–133.

[B3] Grimal P (1996) The Dictionary of Classical Mythology. Wiley-Blackwell, Oxford, 624 pp. https://archive.org/details/dictionaryofclas0000grim/page/n3/mode/2up?view=theater

[B4] Hastriter MW (2007) A review of Ascodipterinae (Diptera: Streblidae) of the Oriental and Australasian regions with a description of three new species of *Ascodipteron* Adensamer and a key to the subfamily. Zootaxa 1636: 1–32. 10.11646/zootaxa.1636.1.1

[B5] Hastriter MW, Bush SE (2006) *Maabella* gen. nov. (Streblidae: Ascodipterinae) from Guangxi Province, China and Vietnam with notes on preservation of Ascodipterinae. Zootaxa 1176: 27–40. 10.11646/zootaxa.1176.1.3

[B6] Kimura M (1980) A simple method for estimating evolutionary rate of base substitutions through comparative studies of nucleotide sequences. Journal of Molecular Evolution 16: 111–120. 10.1007/BF01731581 7463489

[B7] Kumar S, Stecher G, Li M, Knyaz C, Tamura K (2018) MEGA X: Molecular Evolutionary Genetics Analysis across computing platforms. Molecular Biology and Evolution 35: 1547–1549. 10.1093/molbev/msy096 PMC5967553 29722887

[B8] Maa TC (1965) Ascodipterinae of Africa (Diptera: Streblidae). Journal of Medical Entomology 1: 311–326. 10.1093/jmedent/1.4.311 14280481

[B9] Maa TC (1971) Studies in batflies (Diptera: Streblidae; Nycteribiidae). Pacific Insects Monograph 28: 1–247.

[B10] Sun H, Ding L, Yan L, Pape T, Zhang D (2021) *Ascodipteron sanmingensis* sp. nov., a new bat fly (Hippoboscidae: Streblid grade) from Fujian, China. Biodiversity Data Journal 9: e64558. 10.3897/BDJ.9.e64558 PMC8087615 33948101

[B11] Sun H, Ding L, Pape T, Zhang D (2022) A New Species of *Ascodipteron* (Diptera: Hippoboscidae) from China Based on Morphology and DNA Barcodes. Insects 13: 1148. 10.3390/insects13121148 PMC9782136 36555058

[B12] Wang X, Zhou R, Lu L, Wang C, Liu Q (2022) A New Record of *Ornithoica aequisenta* and an Updated Checklist of Hippoboscidae, Nycteribiidae, and Streblidae in China. Journal of Medical Entomology 59(3): 1071–1075. 10.1093/jme/tjac012 35388896

